# Notched Long-Period Fiber Grating with an Amine-Modified Surface Nanostructure for Carbon Dioxide Gas Sensing

**DOI:** 10.3390/ma8074535

**Published:** 2015-07-21

**Authors:** Janw-Wei Wu, Chia-Chin Chiang

**Affiliations:** Department of Mechanical Engineering, National Kaohsiung University of Applied Sciences, Kaohsiung 807, Taiwan; E-Mail: cafa95011@gmail.com

**Keywords:** optical fiber sensor, notched long-period fiber grating, carbon dioxide

## Abstract

This paper presents the fabrication and application of a notched long-period fiber grating (NLPFG) with an amine-modified surface nanostructure for carbon dioxide (CO_2_) gas sensing. The NLPFG with the modified surface nanostructure was fabricated by using inductively coupled plasma (ICP) etching with an Ag nanoparticle etching barrier. The experimental results show that the spectra were changed with the CO_2_ gas flow within 12 min. Thereafter, the spectra of the NLPFG remained steady and unchanged. During the absorption process, the transmission loss was decreased by approximately 2.019 dB, and the decreased rate of transmission loss was 0.163 dB/min. The sensitivity was about −0.089 dB/%. These results demonstrate that the NLPFG CO_2_ gas sensor has the advantages of steady performance, repeatability, and low cost. Therefore, the NLPFG can be utilized as a reliable CO_2_ gas sensor.

## 1. Introduction

Optical fiber sensors have many advantages, such as immunity to electromagnetic interference, low power consumption, corrosion resistance, high temperature resistance, and being lightweight. As a result, optical fiber sensors are widely used in various applications [1]. Long-period fiber grating (LPFG) couples a core mode to a cladding mode. This coupling phenomenon causes a resonant dip in the spectrum, which is very sensitive to external environmental conditions. Thus, LPFG is suitable for use as a sensor. The manufacturing methods for LPFG include excimer laser writing [2], CO_2_ laser writing [3], arc discharged fabrication [4], the mechanical pressure method [5], and fabrication by MEMS technology with etching [6] and photoresist [7]. In this paper, the proposed notched long-period fiber grating (NLPFG) sensor was manufactured by the inductively coupled plasma (ICP) etching method, which was used to create corrugated surface structures on the optical fiber [8].

Various techniques for detecting gas have been developed for applications such as environment monitoring [9], medical diagnosis [10], chemical and mining safety [11], and modern gas laser designs [12]. Among these techniques are several effective optical methods for detecting the gas, including interferometry [13], absorption spectroscopy [14], surface plasmon resonance [15], fluorescence spectroscopy [16], and grating-based refractive index transmission [17].

Around the world in recent years, CO_2_ emissions have been increasing, causing global weather anomalies and the greenhouse phenomenon. Therefore, the production of a CO_2_ gas-monitoring sensor is very important. In 2010, X. Wei *et al.* [18] proposed the perovskite-type metal oxide BSCF (Ba_0.5_Sr_0.5_Co_0.8_Fe_0.2_O_3−δ_) as a gas-sensing layer for coating an LPFG sensor. The perovskite structure of the metal oxide allows CO_2_ to be detected at high temperatures. The absorption effect is best demonstrated in the high temperature range of 600–700 °C and under higher CO_2_ partial pressure. The perovskite structure of the metal oxide after CO_2_ adsorption can be recovered at temperatures higher than 680 °C. At a temperature of 600 °C, the sensor was exposed to a gas mixture containing 10% CO_2_ and 90% air. The resonance wavelength of the LPFG started to decrease, taking approximately 24 min to reach a stable value. At 700 °C, the sensor demonstrated a faster response time and a larger wavelength shift. In 2013, B.N. Shivananju *et al.* [19] proposed clad etched fiber Bragg grating (FBG) with polyallylamine-amino-carbon-nanotube film coated on the surface of the core for detecting CO_2_ gas concentrations. This core FBG sensor for CO_2_ sensing had a response time of 3.07 min. A Bragg wavelength shift of ~6 pm was observed when the CO_2_ gas concentration reached 1000 ppm, and the sensitivity was 1.954 pm/min. The limit of detection calculated was approximately 75 ppm. In 2014, Luis Melo *et al.* [20] proposed a CO_2_ gas concentration sensor comprising a long period grating coated with polystyrene, with coating thicknesses of 204 nm, 249nm, and 365 nm. The sensor had a grating period of 450 µm and a length of 10 mm. The results showed the coating thickness of 365 nm indicated the highest grating sensitivity at approximately 1.23 ± 0.08 pm/% CO_2_. The approaches used by the aforementioned research teams mostly used a sensitive coating layer on the optical fiber sensor, which was suitable for sensing under high temperatures.

This study proposes the use of amine-modified adsorbents as a coating for an NLPFG sensor with a surface nanostructure for CO_2_ gas sensing. The environmental refractive index changes in the sensor are caused by amine-modified adsorbents with CO_2_ capture. Therefore, we can evaluate the spectra change as the test chamber is loaded with the CO_2_ gas. The proposed NLPFG sensor utilizes its nanostructure to improve the function of the amine-modified adsorbents for improved sensitivity.

## 2. Working Principle of the NLPFG Gas Sensor

The NLPFG bears periodic surface-corrugated gratings. As an external load is applied to it, the strain field in the longitudinal direction of the NLPFG is modulated as a square wave because of the periodic surface grating structure of the optical fiber. Based on the elastic-optic effect [21], the refractive index of the NLPFG will also be modulated as a periodic square wave distribution along the optical fiber.

When light is transmitted in the NLPFG, the periodic refractive index grating structure generates a resonant attenuation dip in the spectrum based on the coupled mode theory [7,21]. The resonant attenuation dip (transmission loss) is calculated as

(1) $$T=\cos^{2}(\kappa_{co-cl}^{ac}L)$$

where *L* indicates the length of the LPFG and $\kappa_{co-cl}^{ac}$ is the AC component of the coupling coefficient between the core and cladding modes. The transmission loss of an LPFG can be deduced from the AC component of the coupling coefficient between the core and the cladding. Transmission loss is a function of $\kappa_{co-cl}^{ac}$, which is proportional to the amplitude of changes in the refractive index. From the above formula, it can be seen that the transmission loss of an LPFG is related to the coupling coefficient and grating length. Therefore, the loss can be altered by the external refractive index. In this study, the tetraethylenepentamine (TEPA)-modified adsorbents coat the optical fiber for CO_2_ gas sensing. As the CO_2_ reacts with the sensing layer, the coupling coefficient and effective refractive index are changed. Therefore, the spectra of the NLPFG sensor are deformed and the dips of the spectra are changed. We can measure the CO_2_ by monitoring the transmission loss of the NLPFG.

## 3. Experiment

### 3.1. Production Process and Fabrication of the NLPFG

**Figure 1:**
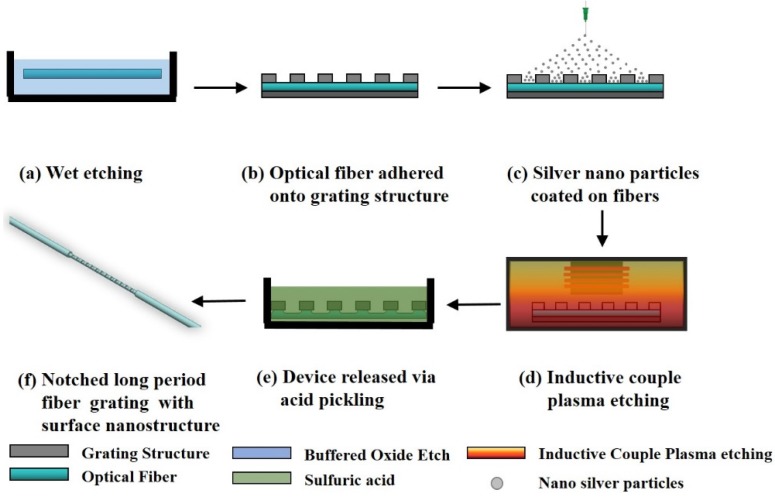
The fabrication process of the notched long-period fiber grating (NLPFG) sensor with the surface needle nanostructure.

**Figure 2:**
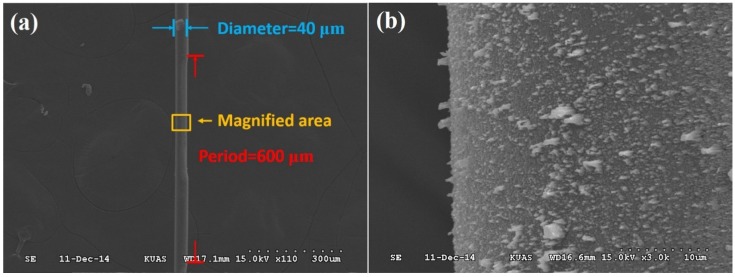
(**a**) SEM image of the NLPFG sensor with the surface needle nanostructure; (**b**) High-magnification SEM images of the NLPFG sensor with the surface needle nanostructure.

In this study, single-mode optical fibers (Corning SMF-28e) were used for fabricating the NLPFG through the ICP dry etching process. First, a buffered oxide etching chemical was employed for etching the fibers in order to reduce the thickness of the fibers from 62.5 to 20 μm. The fibers were attached to a period-structured metal-plated gratings mask (amplitude mask), and, utilizing electrospinning technology, nano-sized silver particles were sprayed onto the fibers as a barrier layer. The gap region between the Ag nano particles was then etched to form the surface nano-needle structure. The nano-needle structured and notched long-period gratings were produced by ICP etching. The nano-needle structure was used mainly to increase the effective area of the sensing layer. The production process is illustrated in Figure 1. The metal-plated gratings mask was designed with periods of 600 μm to achieve a wavelength with a resonant dip close to 1550 nm. The surface-notched period structure was etched on the etched fiber at an ICP etching rate of approximately 2.5 μm/min. Finally, the etched device was released via acid pickling with sulfuric acid (H_2_SO_4_) to remove the high temperature–resistant adhesive on the fiber. The NLPFG with the surface needle nanostructure was thus obtained after it was released from the metal-plated mask. Figure 2 shows a scanning electron microscopy (SEM) image of the NLPFG sensor with the surface nanostructure.

### 3.2. Preparation of the NLPFG Gas Sensor Chip

First, we applied an axial pre-load (0.1 N) on the NLPFG sensor to form a periodic refractive index change for producing a long-period grating effect. Then, ultraviolet adhesive was used to fix the sensor onto a glass plate and thereby prevent any strain from influencing the NLPFG sensor.

### 3.3. Coating the Sensing Layer with Amine-Modified (TEPA-Coated) Adsorbents

A surface modification method was used to coat the NLPFG sensor with amine-modified (TEPA-coated) adsorbents. In order to ensure that the TEPA-modified adsorbent sensing layer was tightly adsorbed on the NLPFG sensor, we used a portable corona treater at a high voltage of 10,000–48,000 V to change the hydrophobic material into hydrophilic material, which ensured that the powder would be strongly attached to the NLPFG sensor. When carbon dioxide was adsorbed by the NLPFG gas sensor, the effective refractive index of the sensor’s cladding was changed. These changes subsequently result in variations in transmission loss. We can measure the carbon dioxide by monitoring the transmission loss of the NLPFG.

### 3.4. The Experimental Setup for the CO_2_ Gas Sensing

**Figure 3:**
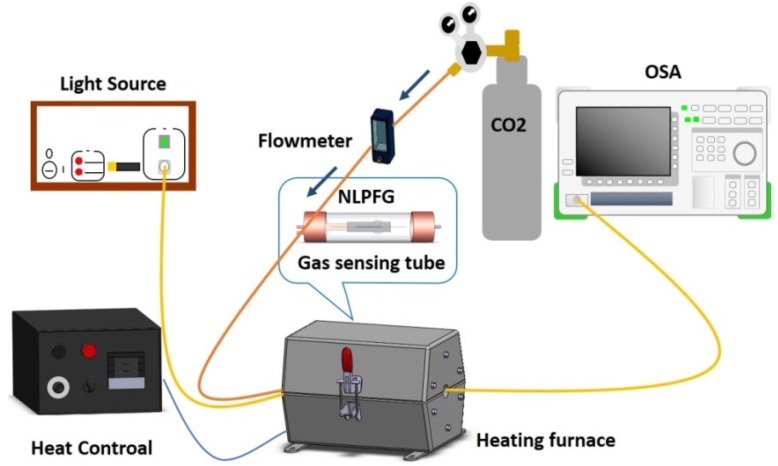
Experimental setup for CO_2_ gas sensing.

Figure 3 shows the experimental setup for the CO_2_ gas sensing. First, we put a prepackaged gas-sensing chip into a gas-sensing tube with amine-modified (TEPA-coated) surfaces. The light source was a super luminescent diode, and the light signal was observed by using an optical spectrum analyzer. The gas used was 15% mixed CO_2_ gas (CO_2_ 15% + N_2_ 85%), which was injected at a flow rate of 0.2 L/min into the gas-sensing tube. The experimental temperature was controlled at room temperature (25 °C). When the CO_2_ reacted with the TEPA-modified adsorbent sensing layer, the refractive index changed. The optical signal change caused by the CO_2_ capture was then observed. The chemical formula for CO_2_ adsorption is indicated below:

CO_2_ + 2R**–**NH_2_ ⟶ RNH_3_^+^ + R–NHCOO^−^

The mechanism of chemical adsorption between the amine active sites and the CO_2_ in anhydrous conditions was the formation of ammonium carbamate [22].

## 4. Results and Discussion

### 4.1. CO_2_ Gas-Sensing Experimental Results

**Figure 4:**
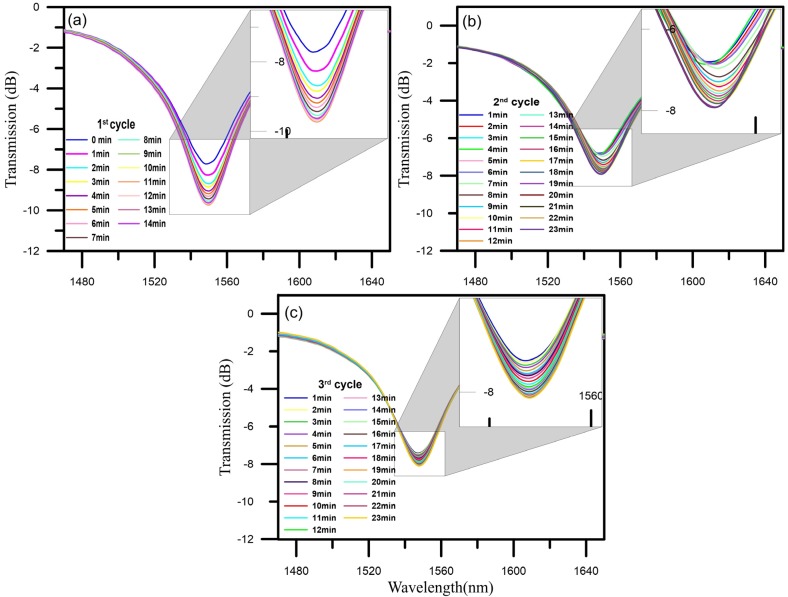
The spectra of CO_2_ gas sensing. (**a**) First cycle spectra of CO_2_ gas sensing; (**b**) Second cycle spectra of CO_2_ gas sensing; (**c**) Third cycle spectra of CO_2_ gas sensing. The insets represent magnified images of the spectra.

The CO_2_ gas-sensing experiments were conducted by using TEPA-modified adsorbents for CO_2_ capture. The experimental results are shown in Figure 4. The resonant wavelengths of the NLPFG (with a period of 600 μm and with a fiber diameter of 40 μm) were 1548.102 nm, and the transmission loss was −18.827 dB. After the addition of the TEPA-modified adsorbent coating to the NLPFG using the dip-coating method, the refractive index changed. This resulted in a drop in the transmission loss to −7.718 dB, so the transmission loss was reduced by −10.109 dB, and the wavelength was shifted by 0.25 nm. This demonstrates that coating with TEPA-modified adsorbents can change the refractive index to influence the magnitude and wavelength position of the attenuated dip. The experimental monitoring was conducted at 1-min intervals. The resonant wavelength shifted slightly, while the transmission loss decreased by approximately 2.019 dB (from −7.718 to −9.737 dB), and the decreased rate of transmission loss was 0.163 dB/min. The CO_2_ sensors reached saturation within 12 min. The spectra of the NLPFG were steady. Therefore, the proposed NLPFG gas sensor can successfully monitor CO_2_ adsorption.

### 4.2. CO_2_ Gas-Sensing Cyclic Adsorption/Desorption Test

The desorption process consisted of heating the NLPFG sensors to 100 ℃ by using a furnace. This temperature was held steady for 20 min before the chamber was cooled to room temperature to allow for the renewal of the gas sensor through desorption of the carbon dioxide. Once the desorption process was complete, the NLPFG gas sensor transmission loss returned to its original value. Figure 4b shows the spectra of the second CO_2_ gas-sensing experiment. The original value of the transmission loss was −6.859 dB. The experimental monitoring was conducted at 1-min intervals. The resonant wavelength shifted slightly, while the transmission loss increased by approximately 1.067 dB (from −6.859 to −7.926 dB). The decreased rate of transmission loss was 0.069 dB/min, and the CO_2_ sensors reached saturation within 21 min. The spectra of the NLPFG remained steady.

**Figure 5:**
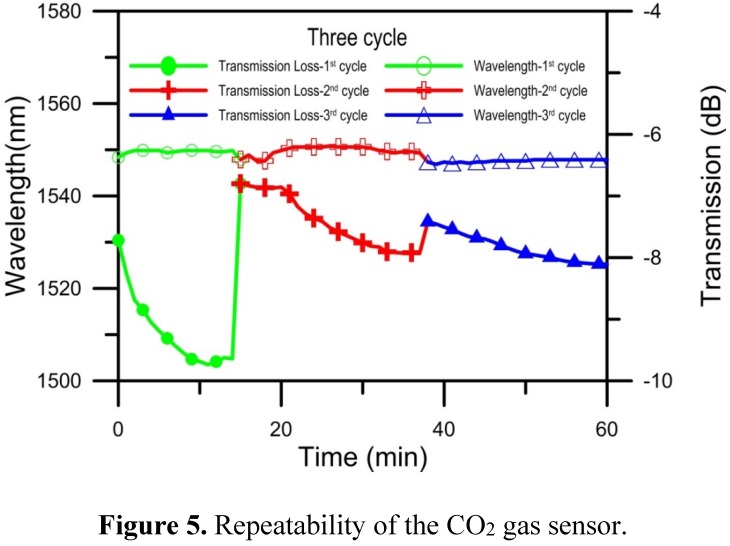
Repeatability of the CO_2_ gas sensor.

We used the same method for the third experiment. The results of the gas sensing are shown in Figure 4c. The original value of the transmission loss of the gas sensor, −7.417 dB, was restored. The experimental monitoring time was unchanged. The resonant wavelength shifted slightly, the transmission loss increased by approximately 0.688 dB (from −7.410 to −8.098 dB), and the decreased rate of transmission loss was 0.036 dB/min. The results of a cyclic adsorption/desorption comparison are shown in Figure 5. The NLPFG CO_2_ gas sensor has the capacity for repeated use, and the required recovery time was short. From the above results, it can be concluded that the NLPFG gas sensor has potential for sensing CO_2_ gas.

### 4.3. CO_2_ Gas Concentrations Sensing Test

**Figure 6:**
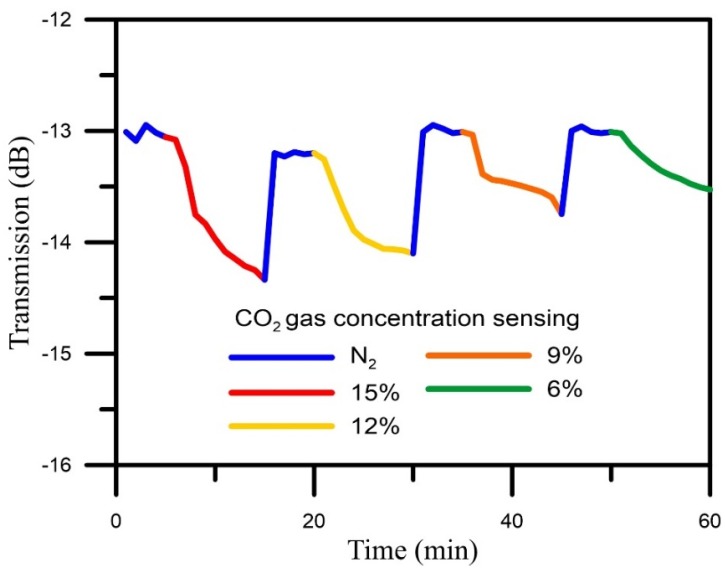
The curve of CO_2_ gas sensing with four different concentrations.

The NLPFG sensor was subjected to a CO_2_ gas-sensing experiment with four different concentrations of CO_2_ (6%, 9%, 12%, 15%). The experimental results are shown in Figure 6. The sensitivity of the NLPFG sensor was about −0.089 dB/%.

## 5. Conclusions

This paper presents a nanostructured amino-modified NLPFG for gas sensing. The results showed that the spectra changed within 12 min and then reached a steady state. This phenomenon indicates the saturation adsorption of the TEPA-modified adsorbents. The transmission loss variation was approximately 2.019 dB (from −7.718 to −9.737 dB), and the decreasing rate of transmission loss was 0.163 dB/min. The sensitivity was about −0.089 dB/%. The cyclic adsorption/desorption experimental results showed that the required recovery time was short. Therefore, the proposed NLPFG gas sensor has potential as a gas sensor for monitoring the CO_2_ adsorption process.
